# Retraction: Regulating BMI1 expression via miRNAs promote Mesenchymal to Epithelial Transition (MET) and sensitizes breast cancer cell to chemotherapeutic drug

**DOI:** 10.1371/journal.pone.0341297

**Published:** 2026-01-21

**Authors:** The PLOS One Editors

Following the publication of this article [1], concerns were raised about results presented in Figs 4–7. Specifically:

- The following panels appear to partially overlap:
  - Fig 4A miR-200a and miR-15a
  - Fig 4B miR-200b and miR-15a
  - Fig 5A 48 hrs miR-200b and Fig 5A 48 hrs miR-203
- The Fig 6B Actin panel appears similar to the Fig 7C Actin panel, despite being used to represent different experimental conditions.
- The Material methods section only reports the use of Student’s t-test and does not clarify the statistical approach used for multivariate analysis, whereas this article appears to present multivariate results.

The first author stated that the issues with Figs 4–7 are the result of figure preparation errors. They disagreed with the statistical concerns, stating that although some figures display multiple outcome measurements side-by-side, and some figures show multiple parameters in a single graph, only univariate comparisons were conducted separately for each parameter. The journal remains concerned about the statistical approach reported for this article. The first author stated that the data underlying the published results are no longer available. In the absence of the individual-level and image data for the figures of concern, the above concerns cannot be resolved.

In light of the above concerns, which raise questions about the reliability of the overall data handling and published results, the *PLOS One* Editors retract this article.

NP agreed with the retraction and apologizes for the issues with the published article. KRG, VKKM, ADN, NV, UB, and MPB either did not respond directly or could not be reached.
